# A systematic review of enteric dysbiosis in chronic fatigue syndrome/myalgic encephalomyelitis

**DOI:** 10.1186/s13643-018-0909-0

**Published:** 2018-12-20

**Authors:** S. Du Preez, M. Corbitt, H. Cabanas, N. Eaton, D. Staines, S. Marshall-Gradisnik

**Affiliations:** 1 National Centre for Neuroimmunology and Emerging Diseases, Menzies Health Institute, Griffith University, Gold Coast, Australia; 2 School of Medical Science, Griffith University, Gold Coast, Australia

**Keywords:** Chronic fatigue syndrome/myalgic encephalomyelitis (CFS/ME), Dysbiosis, Microbiome, Systematic review

## Abstract

**Background:**

Chronic fatigue syndrome or myalgic encephalomyelitis (CFS/ME) is an illness characterised by profound and pervasive fatigue in addition to a heterogeneous constellation of symptoms. The aetiology of this condition remains unknown; however, it has been previously suggested that enteric dysbiosis is implicated in the pathogenesis of CFS/ME. This review examines the evidence currently available for the presence of abnormal microbial ecology in CFS/ME in comparison to healthy controls, with one exception being probiotic-supplemented CFS/ME patients, and whether the composition of the microbiome plays a role in symptom causation.

**Methods:**

EMBASE, Medline (via EBSCOhost), Pubmed and Scopus were systematically searched from 1994 to March 2018. All studies that investigated the gut microbiome composition of CFS/ME patients were initially included prior to the application of specific exclusion criteria. The association between these findings and patient-centred outcomes (fatigue, quality of life, gastrointestinal symptoms, psychological wellbeing) are also reported.

**Results:**

Seven studies that met the inclusion criteria were included in the review. The microbiome composition of CFS/ME patients was compared with healthy controls, with the exception of one study that compared to probiotic-supplemented CFS/ME patients. Differences were reported in each study; however, only three were considered statistically significant, and the findings across all studies were inconsistent. The quality of the studies included in this review scored between poor (< 54%), fair (54–72%) and good (94–100%) using the Downs and Black checklist.

**Conclusions:**

There is currently insufficient evidence for enteric dysbiosis playing a significant role in the pathomechanism of CFS/ME. Recommendations for future research in this field include the use of consistent criteria for the diagnosis of CFS/ME, reduction of confounding variables by controlling factors that influence microbiome composition prior to sample collection and including more severe cases of CFS/ME.

## Background

Chronic fatigue syndrome/myalgic encephalomyelitis (CFS/ME) is a complex and disabling illness of unknown aetiology [1]. Patients experience considerable loss in quality of life (QoL) and diverse symptomatology [2]. Notably, these include persistent fatigue, post-exertional malaise, neurocognitive impairment, autonomic dysfunction, recurrent flu-like symptoms, gastrointestinal (GI) disturbances, and genitourinary manifestations [3]. Furthermore, the underlying pathomechansism is yet to be established, and currently no diagnostic test exists [4]. Instead, diagnosis relies on symptom-specific criteria to identify cases of CFS/ME after all relevant differential diagnoses have been excluded. Centers for Disease Control Fukuda Criteria (1994) [5], Canadian Clinical Case Definition (CCC 2003) [6] and International Consensus Criteria (ICC 2011) [3] are primarily used to identify cases.

The Fukuda criteria were the first set of widely accepted clinical criteria developed to formally diagnose CFS/ME. Its defining element relies on self-reported fatigue of a relapsing or persistent nature that is present for a period of six or more consecutive months [5]. Four of eight additional criteria also need to be present to make a diagnosis under these guidelines. The Fukuda criteria use of a broad, non-specific definition to identify cases of CFS/ME results in limitations in identifying cases with more pronounced fatigue, neurological dysfunction and physical debility when compared to revised classifications of CFS/ME [7]. More recently defined criteria, such as CCC and ICC, include post-exertional neuroimmune exhaustion and autonomic, immune and endocrine dysfunction [3, 6], which are not acknowledged in the Fukuda definition of CFS/ME. While GI symptoms have been recognised under CFS/ME definitions too, alteration in the enteric microbiota, a feature of multiple pathological conditions, has never been specially described as a criterion for any of the following case definitions as further investigation is required.

The human microbiota is an extensive community of over 10,000 different microbial species including bacteria, viruses and archaea. These inhabit various anatomical regions, such as the oral cavity, skin, genitourinary or gastrointestinal tract. While microbes are frequently associated with pathology, naturally occurring symbiotic or commensal flora have co-evolved with the human host and have shown beneficial host interaction including involvement in mediating physiological processes necessary for metabolic and immune function as well as digestion and nutrition. Composition of microbial flora is distinct for each person. Each body region itself contains substantial amount of diversity, particularly the gut [8]. Parameters that affect this composition include internal factors such as the genetic background of the host. External environmental and lifestyle factors can also greatly influence the microbiota too, therefore indicating that the ecosystem is a plastic entity and subject to change [9]. Disruption of the integrity or equilibrium of these intricate microbial networks has been implicated in numerous pathological conditions or exacerbation of disease [8].

In particular, the enteric microbiome is well-established as a requirement for the development of the immune system and lymphoid structures [10, 11]. Additionally, interactions between commensal bacteria and immune cells can stimulate repair and proliferation of the intestinal epithelium [12]. Conversely, evidence exists for perturbations in the gut microbiota, known as dysbiosis, being pathogenic, and, more recently, contributing to chronic diseases [13, 14]. Disruptions to this vital ecosystem have been identified in inflammatory bowel disease (IBD) and more systemic syndromes such as obesity [15]. Consequently, this has generated interest in characterising the microbiome in patients presenting with other chronic illnesses including those with more elusive origins like CFS/ME.

Patients with CFS/ME commonly report a post-infectious onset of the condition attributed to pathogens including bacteria, viruses and parasites [16]. While a causative agent has not yet been identified, research has suggested this illness may still be of microbial or viral origin [17]. A prevailing theory regarding the pathomechanism of CFS/ME is an alteration to the gut microbiome, and subsequent altered functioning of the small bowel, which is purported to establish a hyperpermeable or ‘leaky’ gut [18]. Consequently, this permits the translocation of microbes or their components into the bloodstream, thereby inducing a chronic inflammatory immune response and disruption of the nervous and GI systems [19].

The purpose of this systematic review was to assess the existing literature for evidence of gut dysbiosis and whether changes to microbial ecology contributes to the pathomechanism CFS/ME. Consequently, this review may also serve to guide the development of specific clinical criteria as well as more sensitive and specific diagnostic tests and treatments for this illness.

## Methods

### Literature search

Four databases were searched: EMBASE, Medline (EBSCOhost), Pubmed and Scopus. The following terms were systematically searched as full-text and Medical Subject Headings (MeSH) terms: chronic fatigue (which includes chronic fatigue syndrome and myalgic encephalomyelitis) or systemic exertion intolerance and microbiome (which includes microbiota, gut bacteria/flora, intestinal bacteria/flora, enteric bacteria/flora, microbial flora and microflora), commensal or dysbiosis. All search results were limited to publication date since the establishment of the Fukuda criteria (year 1994–2018). The primary search was performed on 22nd February 2018, and the final search was completed on 31st March 2018.

### Inclusion and exclusion criteria

Studies that fulfilled the following criteria represented in their titles or abstracts were eligible for inclusion: (i) studies that were conducted in humans; (ii) studies written in English available as full text through institutional access; (iii) all studies that investigated the bacterial composition of the microbiome in CFS/ME subjects; (iv) CFS/ME diagnosis according to Fukuda (1994), CCC (2003) or ICC (2011); (v) adults aged 18 years and over; (vi) year searched 1994 to the present year to exclude earlier articles prior to establishment of Fukuda criteria; and (vii) journal articles reporting studies based on original research.

Studies were not included in this review if less than two key search terms were not stated in the title or abstract, and if the criteria used to diagnose CFS/ME were unclear following screening of the full-text. Additionally, studies that used other patient groups (e.g. fibromyalgia (FM), IBD) as a comparison to the CFS/ME cohort were excluded as these conditions commonly co-occur with CFS/ME. Duplicate studies, case reports/studies, or review articles and studies not meeting the above inclusion criteria were also excluded. The primary outcome of interest for this review was the composition of the gut microbiome in CFS/ME. Secondary outcomes evaluated were the association of gut microbiota composition with indicators of illness severity, such as QoL, physical activity and psychological wellbeing. Studies were also excluded if they combined CFS/ME with other patient groups [e.g. CFS/ME and irritable bowel syndrome (IBS)]. Although CFS/ME often co-occurs with IBS, the co-occurrence of IBS was excluded to reduce the risk of confounding, as gut microbiota composition is altered in IBS.

### Selection of studies and data extraction

Titles and abstracts for each article were initially screened on the basis of eligibility criteria. Full-text articles and study quality were independently assessed by two review authors for suitability for inclusion in this review. This was later reassessed and confirmed by all other team members. Eligible studies were read, and the relevant data were extracted (Tables 1 and 2) including (i) study design, (ii) CFS/ME case definition, (iii) country, (iv) sample size, (v) age of participants, (vi) sex and percentage of female participants within the group, (vii) illness duration, (viii) body mass index and weight, (ix) method of quantifying the gut microbiome composition and (x) result of investigation and level of statistical significance.

**Table 1:** Quality assessment and scores of included studies using Downs and Black quality checklist

	Reference						
	Armstrong et al. (2016)	Frémont et al. (2016)	Giloteaux et al. (2016)	Mandarono et al. (2015)	Rao et al. (2014)	Sheedy et al. (2010)	Shukla et al. (2009)
1 Objective of the study clearly described	1	1	1	1	1	1	1
2 Outcomes of interest clearly stated	1	1	1	1	1	1	1
3 Patient characteristics clearly described	1	1	1	1	1	1	1
4 Interventions of interest clearly described	–	–	–	–	1	–	–
5 Are the distributions of principle confounders in each group of subjects to be compared clearly described?	0	0	0	0	0	0	0
6 Main findings of the study clearly described	1	1	1	1	1	1	1
7 Does the study provide estimates of random variability in the data	1	1	1	1	1	1	1
8 Have all important adverse events that may be a consequence of the intervention been reported?	–	–	–	–	1	–	–
9 Have the characteristics of patients lost to follow-up been described?	–	–	–	–	1	–	–
10 Have actual probability values been reported for the main outcomes	1	1	1	1	1	1	1
11 Were the subjects asked to participate in the study representative of the entire population from which they were recruited	1	1	0	0	0	1	1
12 Were those subjects who participated representative of the entire population from which they were recruited	1	1	0	0	0	1	1
13 Were the staff, places and facilities where the patients were treated representative of the treatment the majority of patients receive	–	–	–	–	0	–	
14 Was an attempt made to blind study subjects to the intervention they received?	–	–	–	–	1	–	–
15 Was an attempt made to blind those measuring the main outcomes of the intervention	–	–	–	–	0	–	–
16 If any of the results were based on “data dredging”, was this made clear?	0	0	0	0	0	0	0
17 Do the analyses adjust for different lengths of follow-up of patients	–	–	–	–	1	–	–
18 Were the statistical tests used to assess the main outcomes appropriate?	1	1	1	1	1	1	1
19 Was compliance with the intervention reliable?	–	–	–	–	1	–	–
20 Were the main outcome measures used accurate (valid and reliable)?	1	1	1	1	1	1	1
21 Were the patients recruited from the same population?	1	1	0	0	1	1	1
22 Were subjects recruited over the same period of time?	0	0	0	0	0	1	1
23 Were study subjects randomised to intervention groups?	–	–	–	–	1	–	–
24 Was the randomised intervention assignment concealed from both patients and healthcare staff until recruitment was complete and irrevocable?	–	–	–	–	0	–	–
25 Was there adequate adjustment for confounding in the analyses from which the main findings were drawn?	–	–	–	–	0	–	–
26 Were the losses of patient to follow-up taken into account?	–	–	–	–	1	–	–
27 Did the study have sufficient power to detect a clinically important effect where the probability value for a difference being due to chance is less than 5%?	0	0	0	0	0	0	0
Score	73%	67%	47%	47%	56%	80%	80%

**Table 2:** Summary of study characteristics of included studies

Author	Year	Study design	Sample type	Dx	Country	Samples size		Method of analysing microbiome	Quality score
						CFS/ME	Control		
Armstrong et al.	2016	Observational case-control	CFS/ME	Canadian Criteria (2003)	Australia	34 F	25 F	Bacterial culture and MALDI-TOF MS	73% (good)
Frémont et al.	2013	Observational case-control	CFS/ME	Fukuda (1994)	Belgium	Belgian: 15 F, 3 M; Norwegian: 22 F, 3 M	Belgian: 15 F, 4 M; Norwegian: 14F, 3 M	PCR amplification and high-throughput sequencing of 16S rRNA genes	67% (fair)
Giloteauc et al.	2016	Observational case-control	CFS/ME	Fukuda (1994)	USA	38 F, 11 M	30 F, 9 M	16S rRNA genes sequenced from faecal samples	47% (poor)
Mandarano et al.	2018	Observational case-control	CFS/ME	Fukuda (1994)	USA	Taxa abundance comparisons: 13 F, 4 M; diversity subgroup: 7 F, 4 M	Taxa abundance comparisons: 16 F, 1 M; diversity subgroup: 9 F, 1 M	DNA extraction, 18S amplification, sequencing using QIIME	47% (poor)
Rao et al.	2009	RCT, pilot study	CFS/ME	Canadian Criteria (2003)	Canada	CFS/ME 27 F, 8 M: 16 placebo, 19 treatment (*Lactobacillus casei* strain Shirota)		Culture technique	56% (fair)
Sheedy et al.	2009	Observational case-control	CFS/ME	Holmes (1988) Fukuda (1994) Canadian Criteria (2003)	Australia	108	177	Culture technique	80% (good)
Shukla et al.	2015	Observational case-control	CFS/ME	Fukuda (1994)	Italy	8 F, 2 M	8 F, 2 M	16S rRNA amplification and pyrosequencing	80% (good)

*Dx* diagnostic criteria, *CFS*/*ME* chronic fatigue syndrome/myalgic encephalomyelitis, *F* female, *M* male, *MALDI*-*TOF MS* matrix-assisted laser desorption ionisation time-of flight mass spectrometry, *PCR* polymerase chain reaction, *RNA* ribonucleic acid, *USA* United States of America, *DNA* deoxyribonucleic acid, *QIIME* Quantitative Insights Into Microbial Ecology bioinformatics program

### Quality assessment

The Downs and Black checklist was used to assess study quality and bias [20]. Score ranges have previously been categorised as excellent, good, fair or poor to assist interpretation of scores [21]. For the purposes of this systematic review, these have been converted to percentage ranges, as items for assessing study quality were not consistently relevant across all studies included. The scores are as follows: excellent (94–100%), good (72–94%), fair (54–72%) and poor (< 54%).

## Results

### Overview of studies and study quality

**Fig. 1:**
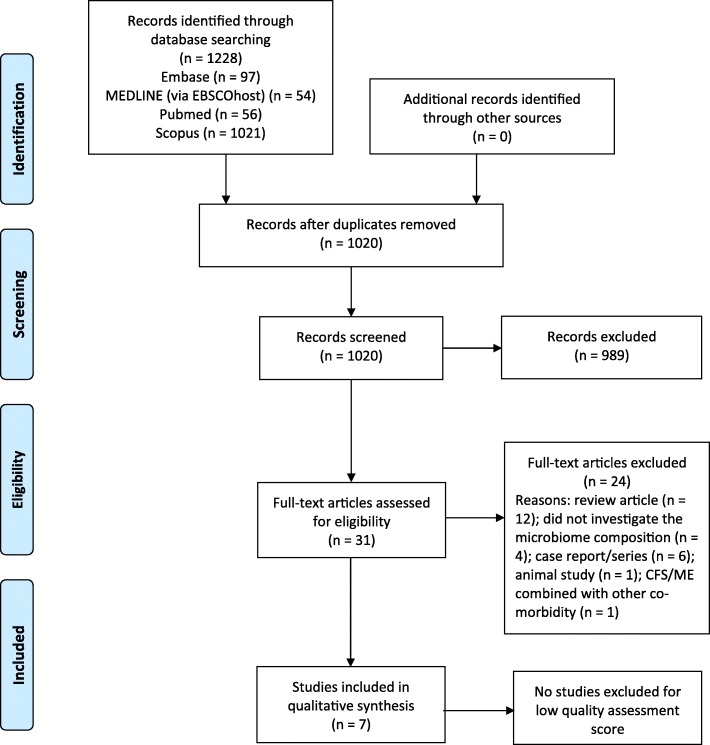
PRISMA flow diagram of literature search for included studies in this review of microbiome composition in CFS/ME

Figure 1 presents the PRISMA flow diagram with the number of included and excluded studies. A total of seven studies were included in this systematic review of the microbiome composition in CFS/ME patients and are summarised in Table 2. The included studies were one randomised control trial (RCT) and six observational cohort studies/case control studies. Studies varied in study quality, with the Downs and Black checklist scores ranging from 47 to 80% (Table 1). Of the included studies, three out of seven were scored as good quality (72–94%) [22–24] and two were scored as fair quality (54–72%) [25, 26]. The remaining two were scored as poor quality (< 54%) [27, 28].

### Participant and study characteristics

The participant characteristics of the included studies are summarised in Tables 2 and 3. Diagnosis of CFS/ME was made using the Fukuda (1994) case definition in four studies [22, 25, 27, 28] and the CCC (2003) in two studies [24, 27]. The remaining study used both Fukuda and CCC as well as Holmes (1988) [23]. Due to the similarities between the Holmes [29] and Fukuda criteria, in addition to the CCC criterion, this publication was included in this review. The mean sample size for each study was 89 participants. The primary outcome of this review was gut microbiome composition in CFS/ME, which was reported in all seven studies [22–28]. All used patient faecal samples as a proxy for investigating intestinal flora composition. Of these, three used culturing methods [23, 24, 26], with one using matrix-assisted laser desorption ionisation time-of flight mass spectrometry (MALDI-TOF MS) after culturing to identify genera [24], three used 16S ribonucleic acid (RNA) amplification and sequencing [22, 25, 27] to investigate bacterial flora of the gut and one study used 18S deoxyribonucleic acid (DNA) amplification and sequencing [28] for investigating eukaryotes in the microbiome. Secondary outcomes evaluated were GI symptoms, psychological wellbeing, cognitive function, QoL and pain and fatigue scores. Four of the included studies did not investigate secondary outcomes as part of their methodology [23–25, 27]. One study reported GI symptoms using an unknown tool and the Bell’s Disability Scale to measure severity of fatigue and cognitive symptoms [28]; two reported psychological symptoms using the Multidimensional Fatigue Inventory (MFI) and profile of mood states (POMS) [22], or Beck Anxiety Inventory (BAI) and Beck Depression Inventory (BDI) [26]; and one reported cognitive function, QoL measures and pain and fatigue scores using MFI and POMS [22].

**Table 3:** Summary of participant characteristics

Reference	Dx	Sample (*n*)		Age (years, mean (SD)		Sex, female (%)		Illness duration	BMI (kg m^−2^), mean (SD)		Weight (kg), mean (SD)	
		CFS	HC	CFS	HC	CFS	HC		CFS	HC	CFS	HC
Armstrong et al.	Canadian (2003)	34	25	34.9 (1.8)	33.0 (1.6)	100%	100%	NR	24.0 (0.81)	23.0 (0.74)	NR	
Frémont et al.	Fukuda (1994)	Belgian: 18 Norwegian: 17	Belgian: 19 Norwegian: 17	38.5 (13) 41 (12.5)	41 (12.6) 45 (19)	83% 88%	79% 82%	Belgian: 7 ± 4 years Norwegian: 12 ± 9 years	NR		NR	
Giloteaux et al.	Fukuda (1994)	49	39	50.2 (12.6)	45.5 (9.9)	78%	77%	NR	25.5 (4.9)	27.1 (6.1)	NR	
Mandarano et al.	Fukuda (1994)	Taxa abundance comparisons 17 Diversity subgroup 11	Taxa abundance comparisons 17 Diversity subgroup 10	52 (11.9) 54 (11.6)	44.6 (10.9) 43.6 (13.6)	76% 64%	94% 90%	NR	26.8 (4.7) 26.2 (4.9)	27.4 (4.5) 27.7 (5.2)	NR	
Rao et al.	Canadian (2003)	CFS placebo: 16 CFS treatment: 19		NR		77%		NR	NR		NR	
Sheedy et al.	Holmes (1988) Fukuda (1994) Candain (2003)	108	177	NR		NR		NR	NR		NR	
Shukla et al.	Fukuda (1994)	10	10	48.6 (10.5)	46.5 (13.0)	80%	80%	NR	23.9 (4.3)	24.6 (3.3)	68.3 (13.7)	65.5 (8.3)

*Dx* diagnostic criteria, *CFS*/*ME* chronic fatigue syndrome/myalgic encephalomyelitis, *HC* healthy control, *BMI* body mass index, *SD* standard deviation, *NR* not reported

### Assessment of microbiome composition

Of the included studies, six reported differences in the microbiome in CFS/ME compared with healthy control (HC) [22–25, 27, 28]. One study reported changes to the microbiome following administration of a probiotic intervention compared with a placebo control as part of a double-blind RCT [26] (Table 4). The following significant differences were reported with respect to CFS/ME patients relative to control subjects: increased *Clostridium* spp. (*p* = 0.020), and decreased total bacteria (*p* = 0.005), total anaerobic bacteria (*p* = 0.021) and *Bacteroides* spp. (*p* = 0.009, *Bacteroides vulgatus* and *Bacteroides uniformis* (not significant) [24]; increased *Lactinofactor* (*p* < 0.001) and *Alistipes* (*p* < 0.05), and decreased *Roseburia* (*p* < 0.05), *Syntrophococcus* (*p < 0.05*), *Holdmenia* (*p* < 0.01) and *Dialister* (*p* < 0.05) in the Norwegian population, and increased *Lactinofactor* (*p* < 0.01) and decreased *Asaccharobacter* (*p* < 0.05) in the Belgian population [25]; reduced phylogenetic diversity (*p* = 0.004) [27]; increased total aerobes (*p* < 0.001), *Enterococcus faecalis* (*p* < 0.001), *Streptococcus sanguinis* (*p* < 0.001) and gram-negative species (*p* < 0.01) [23]; and decreased mean relative abundance of *Actinobacteria* (*p* < 0.05) [22]. Although other differences were reported in these studies, not one was statistically significant.

**Table 4:** Findings into difference in gut microbiome composition between CFS/ME and health controls

Reference	Increased microbial genera in CFS/ME vs HC	Decreased microbial genera in CFS/ME vs HC
Armstrong et al.	*Clostridium* spp. (relative count, *p* = 0.020)	Total bacteria (absolute count, *p* = 0.005), total anaerobic bacteria (absolute count, *p* = 0.021), *Bacteroides* spp. (absolute count, *p* = 0.009; *B*. *vulgatus* and *B. uniformis* not significant)
Frémont et al. *p < 0.05 **p < 0.01	NC vs. BC: *Roseburia* (× 1.7*), *Holdmenia* (× 3**)—Norwegians higher *Firmicutes*	NC vs. BC: *Bacteroides* (× 0.36*), *Alistipes* (× 0.2**), *Barnesiella* (× 0.2**), Parabacteroides (× 0.26**), *Prevotella* (× 0.025**)
	NP vs. NC: *Lactinofactor* (× 20**), *Alistipes* (× 3.8*)	NP vs. NC: *Roseburia* (× 0.54*), *Syntrophococcus* (× 0.4*), *Holdmenia* (× 0.02**), *Dialister* (× 0.6*)
	BP vs. BC: *Lactinofactor* (× 45**)	BP vs. BC: *Asaccharobacter* (× 0.25*)
Giloteaux et al.	Increased pro-inflammatory species, *Proteobacteria* (8%) family *Enterobacteriaceae* (6 vs. 3%) Note: reported ‘overall microbial composition for ME/CFS and controls differed at the phylum and family levels, although none of these were statistically significant after multiple test correction	Reduced phylogenetic diversity (*p* = 0.004) and relative abundance of *Firmicutes* (35%); reduced diversity overall; decreased anti-inflammatory species
Mandarano et al.	Note: investigated Eukaryotes in gut microbiome Composition of Eukaryotic microorganisms was unique between individuals; differences in abundances of specific eukaryotes between CFS/ME and HC did not reach statistical significance at any level of taxonomy Gut eukaryote diversity was not different between CFS and HC	
Rao et al.	Treatment vs. Placebo–treatment was 24 billion CFU *Lactobacillus casei* strain Shirota Moderate increases in total aerobes + anaerobes, significant increases in *Bifidobacteria* and *Lactobacillus* (significance not reported) between treatment and placebo groups from 0 to 8 weeks Note: these results were expected because the probiotics administered contained high levels of these bacteria	
Sheedy et al.	Increased total aerobes (*p* < 0.001), increased *E*. *faecalis* (*p* < 0.001), increased *S*. *sanguinis* (*p* < 0.001)	Lower gram positive to gram negative ratio (*p* < 0.01), decreased total *E*. *coli* (*p* = 0.98)
Shukla et al.	None	Mean relative abundance of *Actinobacteria* decreased (*p* < 0.05), no other significant changes

*CFS*/*ME* chronic fatigue syndrome/myalgic encephalomyelitis, *HC* healthy control, *NC* Norwegian control, *BC* Belgian control, *NP* Norwegian patient, *BP* Belgian patient

### Association of gut microbiome observations on secondary outcomes

From the seven studies, three investigated secondary outcomes on one or more of the following parameters: GI symptoms, psychological wellbeing, cognitive functioning, QoL and pain and fatigue scores (Table 5). One study reported that increased levels of *Bifidobacteria* and *Lactobacillus* in faecal samples of CFS/ME patients receiving 8 weeks of probiotic supplementation were associated with a significant improvement in anxiety scores (*p* = 0.01), but not depression scores. [26]. The other study observed a decrease in the mean relative abundance in *Actinobacteria* in CFS/ME patients, which corresponded with higher MFI scores in CFS/ME vs. HC (*p* < 0.05), particularly greater general and physical fatigue, reduced activity and motivation and greater mental fatigue [22]. These findings may act as a possible link between microbiome composition and CFS/ME symptom severity and other outcomes.

**Table 5:** Secondary outcomes of interest from studies

Author	Secondary outcome measure (s)	Result (s)
GI symptoms		
Mandarono et al.	Unknown tool	Gastrointestinal symptoms reported in 65% CFS/ME vs. 35% HC
Symptom severity		
Mandarano et al.	Bell’s disability scale	Higher reported severity of CFS/ME-related symptoms in CFS/ME vs. HC
QoL		
Shukla et al.	MFI	Higher scores in CFS/ME vs. HC (*p* < 0.05) for general and physical fatigue, reduced activity and motivation and mental fatigue
Psychological symptoms		
Rao et al.	BAI	Improved anxiety scores in the treatment group (*p* = 0.01)
Shukla et al.	POMS	Higher scores for fatigue, confusion and total mood disturbance in CFS/ME vs. HC (*p* < 0.05) Lower scores for vigour in CFS/ME vs. HC (*p* < 0.05)

*GI* gastrointestinal, *IBS* inflammatory bowel syndrome, *CFS*/*ME* chronic fatigue syndrome/myalgic encephalomyelitis, *HC* healthy control, *QoL* quality of life, *MFI* Multidimensional Fatigue Inventory, *BAI* Beck’s Anxiety Inventory, *POMS* Profile of Mood States

## Discussion

Evidence for immunological aberrations in CFS/ME suggests that the underlying pathomechanism may be due to enteric dysbiosis [30]. The proposed mechanism describes an alteration in the mucosal barrier function of the gut, which subsequently becomes hyperpermeable and allows increased translocation of commensal bacteria and their components into the bloodstream, potentially triggering a systemic chronic inflammatory immune response [31]. However, the focus of this systematic review was not to characterise the immune profiles in CFS/ME patients. Rather, to summarise the evidence currently available on the composition of the gut microbiome in patients with CFS/ME compared with the general population, and whether changes in gut microbiota composition are linked with CFS/ME symptoms, including GI symptoms, psychological wellbeing, cognitive function, QoL and pain and fatigue scores. Inclusion and exclusion criteria used to identify the studies examined in this paper were consistent with those used in other systematic reviews [4, 32].

Although diet and medications are known to influence the composition of the microbiome [33–35], this review did not control for these. This is because this would have required alterations to the microbiome by extrinsic factors to also be assessed, which is not the focus of this review. Furthermore, excluding studies that did not control for these parameters would have limited available studies that could be examined to less than seven. However, four of the seven papers included in this review reported attempts to control extrinsic factors prior to sample collection [22–25]. Specifically, three of these papers instructed participants to cease anti-microbial and probiotic agents between 2 and 4 weeks prior to faecal collection [23–25]. Moreover, Shukla et al. excluded participants if they reported current use of antibiotics or probiotics in addition to laxatives, stool softeners and anti-diarrheal agents. Additionally, Fremont et al. reported results involving CFS/ME patients and HC from different geographical regions. Their study demonstrated that geographical origin influenced gut microbiome composition between Norwegian and Belgian participants. Thus, extrinsic and intrinsic factors may also be influenced by geographical region. Overall, the present review elucidates the methodological limitations in current literature and an insufficiency of evidence to establish a link between gut dysbiosis and the pathomechanism of CFS/ME or any of the symptoms exhibited.

### Participants and study characteristics

The present review was comprised primarily of female participants ageing between 35 and 54 years. These findings are consistent with epidemiological studies of CFS/ME that report a greater prevalence of the illness in females and those aged 35 and 45 years [16, 36, 37]. The participants resided in Australia, Europe or North America, and one of the seven publications included information regarding the ethnicities of participants [25]. Moreover, most of the studies examined in the current review used the Fukuda (1994) case definition [5]. The broad nature of the Fukuda case definition may have contributed to the inconsistency of the findings generated by studies in the present review to identify notable alterations to the microbiome of CFS/ME patients. Revised criteria after Fukuda (1994), such as CCC (2003) and ICC (2011), better defined the illness that characteristically presents with a diverse array of symptoms and creates consistency with CFS/ME diagnosis. Similarly, not all studies used the same case definition of CFS/ME, making it difficult to compare findings due to the inherent differences between the criteria.

A number of methods were used to identify microbiota alterations in the gut, with no two studies in this review using identical methods of investigating the microbiome. This finding suggests that there is currently no standardised protocol for investigating the composition of the microbiome, which limits the ability to make appropriate comparisons between studies. Furthermore, the instruments to measure secondary outcomes (e.g. fatigue, psychological symptoms) were inconsistent between the studies that considered these parameters.

Although the studies recruited patients through clinics, universities or hospitals, none reported the severity spectrum of illness presentation. Due to the nature of recruiting, many of these studies may have excluded severely affected CFS/ME patients that were rendered house- or bed-bound due to their inability to attend an out of home location for screening or sample collection. This potential sampling bias is likely to confound the results and limit the validity of the findings to only patients with mild or moderate illness severity with the capacity to venture outside their homes. It is therefore essential for future research to consider the varying severity present in CFS/ME when designing and implementing research methodology to accommodate and include all representations of this illness under a range of settings (e.g. in a clinic or patient’s home).

### Microbiome composition

CFS/ME is complex illness believed to be a multisystemic disorder affecting the immune, nervous, GI, cardiovascular and endocrine systems [38]. The pathomechanism of this condition is yet to be established; however, it has been suggested that CFS/ME is a manifestation of gut dysbiosis [30, 31]. A systematic review published in 2016 on the use of certain drug therapies, which included antibiotics, did not show these therapies to be beneficial in treating CFS/ME [39]. Similarly, a systematic review published earlier this year concluded that probiotics were ineffective in treating CFS/ME [4]. The findings of these systematic reviews suggest that alterations in the gut microbiome do not contribute to the pathomechanism of the illness. Consequently, administration of probiotics or antibiotics as a means to treat CFS/ME is not supported by the evidence.

Each observational study, while using different research methods, reported variability in the gut microbiome composition of CFS/ME patients compared to HC; however, only some studies reached statistical significance. Importantly, all studies included in this review used faecal samples as a proxy to determine gut microbiome composition. The use of stool bacteria may merely represent luminal bacteria and not gut mucosal flora analysed by gastric tissue biopsy [40]. Additionally, the use of culture methods which are less specific than DNA and/or RNA amplification and sequencing techniques may be problematic [41, 42]. Increased total anaerobic bacteria or decreased total aerobic bacteria was described in two studies, both using culturing methods and nuclear magnetic resonance (NMR) spectroscopy [23, 24]. Although increases in *Clostridium* spp. and *Enterococcus faecalis* were reported [23, 24], diets that are high in sugar and high in fat are believed to encourage growth of *Clostridium* and *Enterococcus* spp., among others [43]. These two studies did not control for diet ahead of sample collection, thereby limiting the validity of their findings. This suggests the necessity to control extrinsic factors, such as diet, in addition to utilising more sensitive gut microbiota profiling methods.

The studies that surveyed secondary outcome measures related to the symptoms of CFS/ME when compared with HC reported that CFS/ME patients are more commonly affected by GI disturbances; higher disability, pain and fatigue scores; and reduced emotional wellbeing, motivation and mental functioning [44]. Again, inconsistency in the instruments used to measure these parameters made it difficult to perform valid assessments of the data. A paper not included in this review due to the inclusion of patients with IBS by Nagy-Szakal and colleagues reported on direct correlations between the relative abundance of specific bacterial strains and scores on the MFI and 36-Item Short Form Health Survey (SF-36) questionnaires [45]. The RCT conducted by Rao et al. that administered 24 billion colony forming units *Lactobacillus casei* strain Shirota to the treatment group of CFS/ME reported significant increases in *Bifidobacteria* and *Lactobacillus* compared to the control group after 8 weeks [26].

### Quality assessment

The quality of the studies identified and included in this review ranged from poor to good. The Downs and Black Checklist was used to assess the quality of studies included in this review because it has previously been identified as a reliable tool for assessing both case-control and RCT studies, which have been examined in this systematic review [46]. Furthermore, questions not relevant to the current review from this comprehensive checklist could be eliminated without significantly impacting its ability to differentiate the overall quality of the studies and their findings. Using this tool, we were able to illustrate that the standard of current evidence for the existence of enteric dysbiosis in CFS/ME is inadequate to justify its inclusion as a criterion for diagnosis or basis for treatment.

## Conclusions

The primary aim of this systematic review was to examine the current evidence for alterations in the gut microbiome indicating the pathomechanism of CFS/ME. Additionally, a secondary aim sought to determine whether there were any associations with gut dysbiosis and symptom manifestation in CFS/ME. The findings of our systematic review demonstrate that current evidence is inconsistent, and we are unable to draw any significant link between gut dysbiosis and the pathomechanism of CFS/ME. This emphasises the need for specific clinical criteria to be used when diagnosing the condition in addition to reduction of confounding variables by controlling factors that influence microbiome composition prior to sample collection as well as including more severe cases of CFS/ME. Based on currently available data presented in this systematic review, the effectivity of gastrointestinal flora altering therapy in the treatment of CFS/ME is yet to be confirmed.

## Abbreviations

BAI: Beck Anxiety Inventory
BC: Belgian control
BDI: Beck Depression Inventory
BMI: Body mass index
BP: Belgian patient
CCC: Canadian Clinical Case Definition
CFS/ME: Chronic fatigue syndrome/myalgic encephalomyelitis
DNA: Deoxyribonucleic acid
Dx: Diagnostic criteria
F: Female
FM: Fibromyalgia
GI: Gastrointestinal
HC: Healthy control
IBD: Inflammatory bowel disease
IBS: Irritable bowel syndrome
ICC: International Consensus Criteria
M: Male
MALDI-TOF MS: Matrix-assisted laser desorption ionisation time-of flight mass spectrometry
MeSH: Medical Subject Headings
MFI: Multidimensional fatigue inventory
NC: Norwegian control
NMR: Nuclear magnetic resonance
NP: Norwegian patient
NR: Not reported
POMS: Profile of mood states
QIIME: Quantitative Insights Into Microbial Ecology bioinformatics program
QoL: Quality of life
RCT: Randomised control trial
RNA: Ribonucleic acid
SD: Standard deviation
SF-36: 36-Item Short Form Health Survey
USA: United States of America

## References

[1] Panelli MC (2017). JTM advances in uncharted territories: diseases and disorders of unknown etiology. J Transl Med.

[2] Johnston SC, Brenu EW, Hardcastle SL, Huth TK, Staines DR, Marshall-Gradisnik SM (2014). A comparison of health status in patients meeting alternative definitions for chronic fatigue syndrome/myalgic encephalomyelitis. Health Qual Life Outcomes.

[3] Carruthers BM, van de Sande MI, De Meirleir KL, Klimas NG, Broderick G, Mitchell T (2011). Myalgic encephalomyelitis: international consensus criteria. J Intern Med.

[4] Corbitt M, Campagnolo N, Staines D, Marshall-Gradisnik S. A systematic review of probiotic interventions for gastrointestinal symptoms and irritable bowel syndrome in chronic fatigue syndrome/myalgic encephalomyelitis (CFS/ME). Probiotics and Antimicrobial Proteins. 2018; 10.1007/s12602-018-9397-8. 10.1007/s12602-018-9397-8 29464501

[5] Fukuda K (1994). The chronic fatigue syndrome: a comprehensive approach to its definition and study. Ann Intern Med.

[6] Carruthers BM, Jain AK, Meirleir KLD, Peterson DL, Klimas NG, Lerner AM (2003). Myalgic encephalomyelitis/chronic fatigue syndrome. Journal of Chronic Fatigue Syndrome..

[7] Jason LA, Torres-Harding SR, Jurgens A, Helgerson J (2004). Comparing the Fukuda et al. criteria and the Canadian case definition for chronic fatigue syndrome. Journal of Chronic Fatigue Syndrome.

[8] Liang D, Leung RK-K, Guan W, Au WW. Involvement of gut microbiome in human health and disease: brief overview, knowledge gaps and research opportunities. Gut Pathog. 2018;10 10.1186/s13099-018-0230-4. 10.1186/s13099-018-0230-4 PMC5785832 29416567

[9] Candela M, Biagi E, Maccaferri S, Turroni S, Brigidi P (2012). Intestinal microbiota is a plastic factor responding to environmental changes. Trends Microbiol.

[10] Belkaid Y, Hand T (2014). Role of the microbiota in immunity and inflammation. Cell.

[11] Kamada N, Núñez G (2013). Role of the gut microbiota in the development and function of lymphoid cells. J Immunol.

[12] Hooper LV, Littman DR, Macpherson AJ (2012). Interactions between the microbiota and the immune system. Science.

[13] Cho I, Blaser MJ (2012). The human microbiome: at the interface of health and disease. Nat Rev Genet.

[14] Langdon A, Crook N, Dantas G (2016). The effects of antibiotics on the microbiome throughout development and alternative approaches for therapeutic modulation. Genome Medicine.

[15] Guinane CM, Cotter PD (2013). Role of the gut microbiota in health and chronic gastrointestinal disease: understanding a hidden metabolic organ. Therap Adv Gastroenterol.

[16] Wyller VB (2007). The chronic fatigue syndrome—an update. Acta Neurol Scand.

[17] Afari N, Buchwald D (2003). Chronic fatigue syndrome: a review. AJP.

[18] Maes M, Coucke F, Leunis J-C (2007). Normalization of the increased translocation of endotoxin from gram negative enterobacteria (leaky gut) is accompanied bya remission of chronic fatigue syndrome. Neuroendocrinol Lett.

[19] Maes M, Kubera M, Leunis J-C, Berk M (2012). Increased IgA and IgM responses against gut commensals in chronic depression: further evidence for increased bacterial translocation or leaky gut. J Affect Disord.

[20] Downs SH, Black N (1998). The feasibility of creating a checklist for the assessment of the methodological quality both of randomised and non-randomised studies of health care interventions. J Epidemiol Community Health.

[21] Hooper P, Jutai JW, Strong G, Russell-Minda E (2008). Age-related macular degeneration and low-vision rehabilitation: a systematic review. Can J Ophthalmol.

[22] Shukla SK, Cook D, Meyer J, Vernon SD, Le T, Clevidence D, et al. Changes in gut and plasma microbiome following exercise challenge in Myalgic encephalomyelitis/chronic fatigue syndrome (ME/CFS). PLoS One. 2015;10 10.1371/journal.pone.0145453. 10.1371/journal.pone.0145453 PMC4684203 26683192

[23] Sheedy JR, Wettenhall REH, Scanlon D, Gooley PR, Lewis DP, Mcgregor N (2009). Increased D-lactic acid intestinal bacteria in patients with chronic fatigue syndrome. In Vivo.

[24] Armstrong CW, McGregor NR, Lewis DP, Butt HL, Gooley PR. The association of fecal microbiota and fecal, blood serum and urine metabolites in myalgic encephalomyelitis/chronic fatigue syndrome. Metabolomics. 2017;13 10.1007/s11306-016-1145-z.

[25] Frémont M, Coomans D, Massart S, De Meirleir K (2013). High-throughput 16S rRNA gene sequencing reveals alterations of intestinal microbiota in myalgic encephalomyelitis/chronic fatigue syndrome patients. Anaerobe.

[26] Rao AV, Bested AC, Beaulne TM, Katzman MA, Iorio C, Berardi JM (2009). A randomized, double-blind, placebo-controlled pilot study of a probiotic in emotional symptoms of chronic fatigue syndrome. Gut Pathog.

[27] Giloteaux L, Goodrich JK, Walters WA, Levine SM, Ley RE, Hanson MR. Reduced diversity and altered composition of the gut microbiome in individuals with myalgic encephalomyelitis/chronic fatigue syndrome. Microbiome. 2016;4 10.1186/s40168-016-0171-4. 10.1186/s40168-016-0171-4 PMC4918027 27338587

[28] Mandarano AH, Giloteaux L, Keller BA, Levine SM, Hanson MR. Eukaryotes in the gut microbiota in myalgic encephalomyelitis/chronic fatigue syndrome. PeerJ. 2018;2018 10.7717/peerj.4282. 10.7717/peerj.4282 PMC5784577 29375937

[29] Holmes GP, Kaplan JE, Gantz NM, Komaroff AL, Schonberger LB, Straus SE (1988). Chronic fatigue syndrome: a working case definition. Ann Intern Med.

[30] Brown BI (2014). Chronic fatigue syndrome: a personalized integrative medicine approach. Altern Ther Health Med.

[31] Maes M, Mihaylova I, Leunis J-C (2007). Increased serum IgA and IgM against LPS of enterobacteria in chronic fatigue syndrome (CFS): indication for the involvement of gram-negative enterobacteria in the etiology of CFS and for the presence of an increased gut-intestinal permeability. J Affect Disord.

[32] Campagnolo N, Johnston S, Collatz A, Staines D, Marshall-Gradisnik S (2017). Dietary and nutrition interventions for the therapeutic treatment of chronic fatigue syndrome/myalgic encephalomyelitis: a systematic review. J Hum Nutr Diet.

[33] Lawrence J (2016). Fresh evidence sheds light on chronic fatigue syndrome. Pharmaceutical Journal.

[34] Forslund K, Hildebrand F, Nielsen T, Falony G, Chatelier EL, Sunagawa S (2015). Disentangling type 2 diabetes and metformin treatment signatures in the human gut microbiota. Nature.

[35] Preidis GA, Versalovic J (2009). Targeting the human microbiome with antibiotics, probiotics, and prebiotics: gastroenterology enters the metagenomics era. Gastroenterology.

[36] Collin SM, Crawley E, May MT, Sterne JA, Hollingworth W (2011). The impact of CFS/ME on employment and productivity in the UK: a cross-sectional study based on the CFS/ME national outcomes database. BMC Health Serv Res.

[37] Johnston SC, Staines DR, Marshall-Gradisnik SM (2016). Epidemiological characteristics of chronic fatigue syndrome/myalgic encephalomyelitis in Australian patients. Clin Epidemiol.

[38] Glassford JAG. The neuroinflammatory etiopathology of myalgic encephalomyelitis/chronic fatigue syndrome (ME/CFS). Front Physiol. 2017;8 10.3389/fphys.2017.00088. 10.3389/fphys.2017.00088 PMC5314655 28261110

[39] Collatz A, Johnston SC, Staines DR, Marshall-Gradisnik SM (2016). A systematic review of drug therapies for chronic fatigue syndrome/myalgic encephalomyelitis. Clin Ther.

[40] Momozawa Y, Deffontaine V, Louis E, Medrano JF (2011). Characterization of bacteria in biopsies of colon and stools by high throughput sequencing of the V2 region of bacterial 16S rRNA gene in human. PLoS One.

[41] Morgan f U, Pallant L, Dwyer BW, Forbes DA, Rich G, Thompson RCA (1998). Comparison of PCR and microscopy for detection of Cryptosporidium parvum in human fecal specimens: clinical trial. J Clin Microbiol.

[42] Verweij JJ, Blangé RA, Templeton K, Schinkel J, Brienen EA, van Rooyen MA (2004). Simultaneous detection of Entamoeba histolytica, Giardia lamblia, and Cryptosporidium parvum in fecal samples by using multiplex real-time PCR. J Clin Microbiol.

[43] Brown K, DeCoffe D, Molcan E, Gibson DL (2012). Diet-induced dysbiosis of the intestinal microbiota and the effects on immunity and disease. Nutrients.

[44] Nacul LC, Lacerda EM, Campion P, Pheby D, Drachler M de L, Leite JC (2011). The functional status and well being of people with myalgic encephalomyelitis/chronic fatigue syndrome and their carers. BMC Public Health.

[45] Nagy-Szakal D, Williams BL, Mishra N, Che X, Lee B, Bateman L (2017). Fecal metagenomic profiles in subgroups of patients with myalgic encephalomyelitis/chronic fatigue syndrome. Microbiome.

[46] Sanderson S, Tatt ID, Higgins JP (2007). Tools for assessing quality and susceptibility to bias in observational studies in epidemiology: a systematic review and annotated bibliography. Int J Epidemiol.

